# Breast Cancer Mortality in the Americas and Australasia over the Period 1980–2017 with Predictions for 2025

**DOI:** 10.3390/biology10080814

**Published:** 2021-08-23

**Authors:** Cezary Wojtyla, Paola Bertuccio, Michal Ciebiera, Carlo La Vecchia

**Affiliations:** 1 International Prevention Research Institute—Collaborating Centre, Calisia University, 16 Kaszubska St., 62-800 Kalisz, Poland; 2 Department of Biomedical and Clinical Sciences “L. Sacco”, Università degli Studi di Milano, Via Giovanni Battista Grassi 74, 20157 Milan, Italy; paola.bertuccio@unimi.it; 3 Second Department of Obstetrics and Gynecology, Center of Postgraduate Medical Education, 80 Ceglowska St., 01-809 Warsaw, Poland; michal.ciebiera@gmail.com; 4 Department of Clinical Sciences and Community Health, Università degli Studi di Milano, Vanzetti 5, 20133 Milan, Italy; carlo.lavecchia@unimi.it

**Keywords:** breast cancer, trends, mortality, projections

## Abstract

**Simple Summary:**

Globally, breast cancer is the most common neoplasm and the leading cause of cancer death in women. It is also the common cancer for which the largest advancements have been made in terms of screening, early diagnosis, management and treatment over the last decades. These advances have had an impact on breast cancer mortality, which therefore depends on many aspects, including countries income and the health care system, leading to inequalities across the world. Breast cancer mortality has been substantially decreasing in high income countries of North America and Australia, but trends have been less consistent in Latin America and Asia, indicating the scope for further global advancemets in screening and management of breast cancer.

**Abstract:**

Substantial progress has been made in the diagnosis, management, and treatment of breast cancer over the last decades. This has affected mortality rates but has also led to inequality in epidemiological trends between different regions of the world. We extracted death certification data for breast cancer from the World Health Organization database. We analyzed trends in breast cancer mortality in selected countries from America, Asia, and Oceania over the 1980–2017 period and predicted numbers of deaths and rates for 2025. In North America, we observed decreased breast cancer mortality, reaching a rate of about 13/100,000 women in 2017. In Latin American countries, breast cancer mortality rates did not consistently decrease. The highest decreases in mortality were observed in Australia. Mortality trends in Asian countries remained among the lowest globally. We have predicted decreased mortality from breast cancer in 2025 for most of the analyzed countries. The epidemiological situation regarding breast cancer mortality is expected to change in the coming years. Advancements in diagnosis and treatment of breast cancer must be extended in various areas of the world to obtain global control of breast cancer mortality.

## 1. Introduction

Globally, breast cancer is the most common neoplasm and the leading cause of cancer death in women, with over 2 million cases and about 700,000 deaths per year [1]. It is also the cancer for which the greatest progress has been made in terms of screening, early diagnosis, management, and treatment options over the last decades [2,3]. This has contributed to increasing inequality in patterns and trends of breast cancer between different regions of the world. Currently, lower mortality rates are observed in several high-income countries than in low- and middle-income countries [4,5,6,7].

We observed favorable breast cancer mortality trends in North America and Oceania, as well as in most western European countries [3,8]. At the same time, we predicted a continuation of the observed trend and a further 10% decrease in mortality in the regions of North America and Oceania in 2020.

In this study, we present updated trends in breast cancer mortality in selected countries from America, Asia, and Oceania, as well as our predictions for mortality rates for 2025.

## 2. Materials and Methods

We extracted official death certification data for breast cancer in women for various countries worldwide from 1980 to 2017 (for most countries) or the most recent available calendar year (2018 for a few countries) from the World Health Organization (WHO) mortality database [9]. During the calendar period considered, we used three different Revisions of the International Classification of Diseases (ICD), recoding cancer deaths for each country and calendar year according to the 10th Revision of the ICD (code C50) [10].

We obtained estimates of the resident populations based on official censuses from the same WHO database, and when unavailable from the United Nations (UN) and Pan-American Health Organization (PAHO) databases [11,12].

We selected 14 countries from the Americas and another 8 countries from Asia and Oceania based on the following criteria: countries with over 1 million female inhabitants and a mortality data coverage of over 90%, except for Guatemala (88%) and Brazil (87%).

For each country and calendar year, we computed the age-specific rates for subsequent five-years age groups (from 0–4 to 85+ years and to 80+ for the American countries). Then, we computed the age-standardized mortality rates per 100,000 person-years based on the world standard population [13] for all ages and 4 age groups (20–49, 50–69, 70–79, and 80+).

For a subset of 16 major countries with over 4 million female inhabitants, we carried out a joinpoint regression analysis of mortality trends for breast cancer over the 1980–2017 period to identify possible significant changes [14]. We identified the time point (s), called ‘joinpoints’, where a change in the linear slope (on a log scale) of the temporal trend occurred by testing from zero up to a maximum of five joinpoints. We also estimated the annual percent change (APC) for each of the identified linear segment by fitting a regression line to the natural logarithm of the rates using calendar year as a covariate. We also estimated the average APC (AAPC) over the entire study period as the geometric weighted average of the APC, with the weights equal to the length of each time interval segment [15,16].

We estimated the predicted age-specific numbers of deaths and the corresponding 95% prediction intervals (PIs) for 2025 for the selected 16 major countries. Firstly, we fitted a joinpoint model to the number of certified deaths in each five-year age group, then identified the most recent trend segment. Subsequently, we applied a linear regression to the mortality data in each age group over the time period identified by the joinpoint model. We computed the predicted age-standardized mortality rates with their 95% PIs using the predicted age-specific numbers of deaths and predicted populations from UN and PAHO databases [11,12].

We estimated the number of avoided cancer deaths from breast cancer over the 1991–2025 period by comparing the observed and expected deaths on the basis of the 1990 age-specific peak rates for selected countries, i.e., Argentina, Canada, Chile, the USA, Australia, and New Zealand.

## 3. Results

Table 1 gives the age-standardized mortality rates for breast cancer at all ages in selected American, Asian and Oceanian countries around 2012 (2010–2014 quinquennium) and in 2017 (or the last available year), along with the numbers of deaths of the last available year and percent changes between 2017 and 2012. In North America, we observed a decrease in breast cancer mortality, reaching a rate of about 13/100,000 women in 2017. In Latin American countries, breast cancer mortality rates were more heterogeneous. Decrease rates were observed in Cuba (−10.9%), Chile (−4.9%), Puerto Rico (−4.2%), and Uruguay (−2.8%). Stable mortality rates were observed in Argentina and Panama, while the rest of the countries showed increases in mortality, with percent changes ranging from 1.8% in Costa Rica to 11.3% in Guatemala. The highest decrease in mortality over the analyzed period was observed in Australia (−11.2%), reaching a rate of 11.8/100,000 women in 2017. Mortality rates from breast cancer also decreased in Israel (−6.9%). In Hong Kong, Japan, and Korea, we observed increases in mortality, although the rates in 2017 were among the lowest of all the analyzed countries. Kuwait was the country with the highest increase in mortality from breast cancer (12.9%)—the rate reached 17.1/100,000 women in 2017.

**Figure 1:**
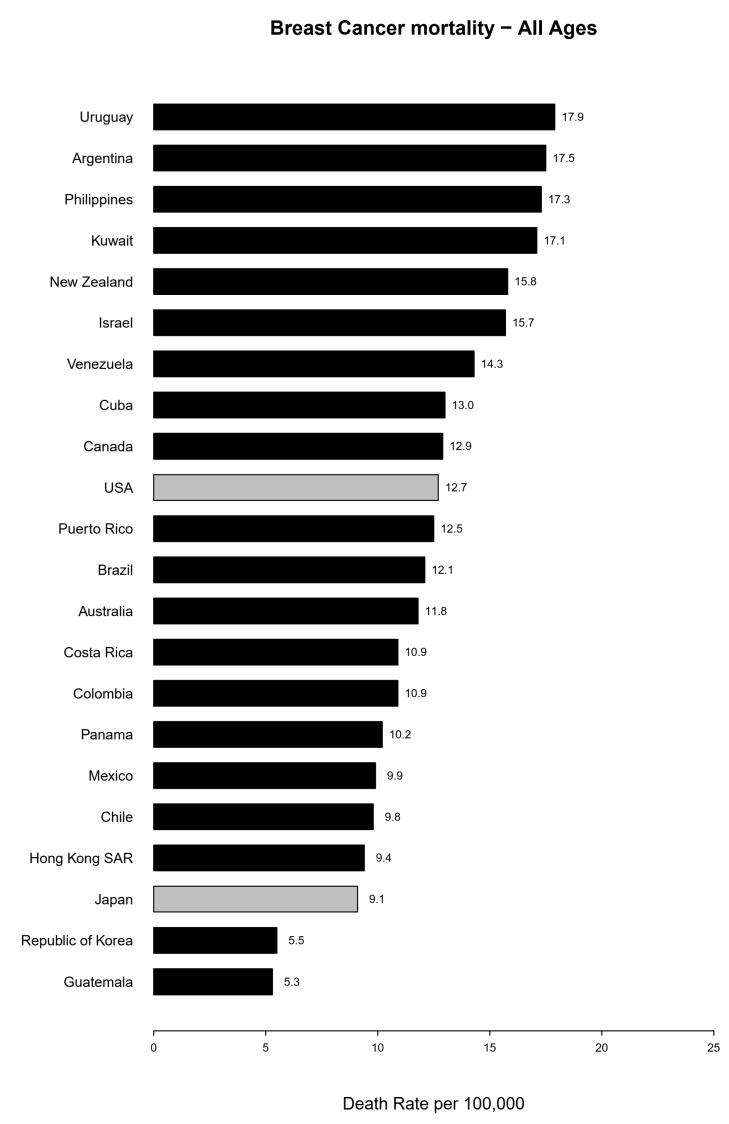
Bar plot of the age-standardized (world population) rates for breast cancer registered in 2017 (or 2015 for Kuwait), ordered from the highest to the lowest rate.

Figure 1 ranks the mortality rates observed in 2017. In 2017, the highest mortality rates were observed in Uruguay (17.9/100,000 women), Argentina (17.5), the Philippines (17.3), and Kuwait (17.1). The lowest rates were reported in Guatemala (5.3/100,000 women), Korea (5.5), Japan (9.1), and Hong Kong (9.4).

Table 2 presents the age-standardized mortality rates for breast cancer in the age ranges of 20–49, 50–69, 70–79, and over 80 years old for selected American and Australasian countries around 2012 (2010–2014 quinquennium) and in 2017 (or the last available year), the numbers of deaths for the latest year, and the corresponding percent changes in the rates between 2017 and 2012. In North America, we observed decreased breast cancer mortality in all age groups, except for the oldest one and the youngest age groups in Canada. Among women aged 20–49 in Latin American countries, mortality rates decreased only in Chile and Cuba. The rest of the countries showed increased mortality rates. In the same age group, we observed decreased mortality rates in Kuwait and Australia. Stable mortality was observed in New Zealand and Japan, while the rest of the Asian and Oceanian countries showed increases in mortality. In women aged 50–69, mortality rates increased in Colombia, Mexico, Brazil, and Hong Kong, while the rates were stable in Chile, Japan, and Korea. The other analyzed countries in this age group showed decreased mortality. In most of the countries in Latin America, Asia, and Oceania, we observed increased mortality from breast cancer among women aged 70–79 years. Stable mortality was observed in Panama, whilst decreases were observed in Australia (−8.3%), Uruguay (−6.8%), and Israel (−2.4%). The greatest heterogeneity was observed among the oldest group of women. In Latin American countries, stable mortality was observed in Mexico, with a slight increase in Brazil (3.4%). The greatest decrease was observed in Chile. Mortality was stable in Australia. Decreased mortality was observed in Hong Kong (−16.5%) and Israel (−3.1%), whilst the rest of the countries showed increased mortality.

**Figure 2:**
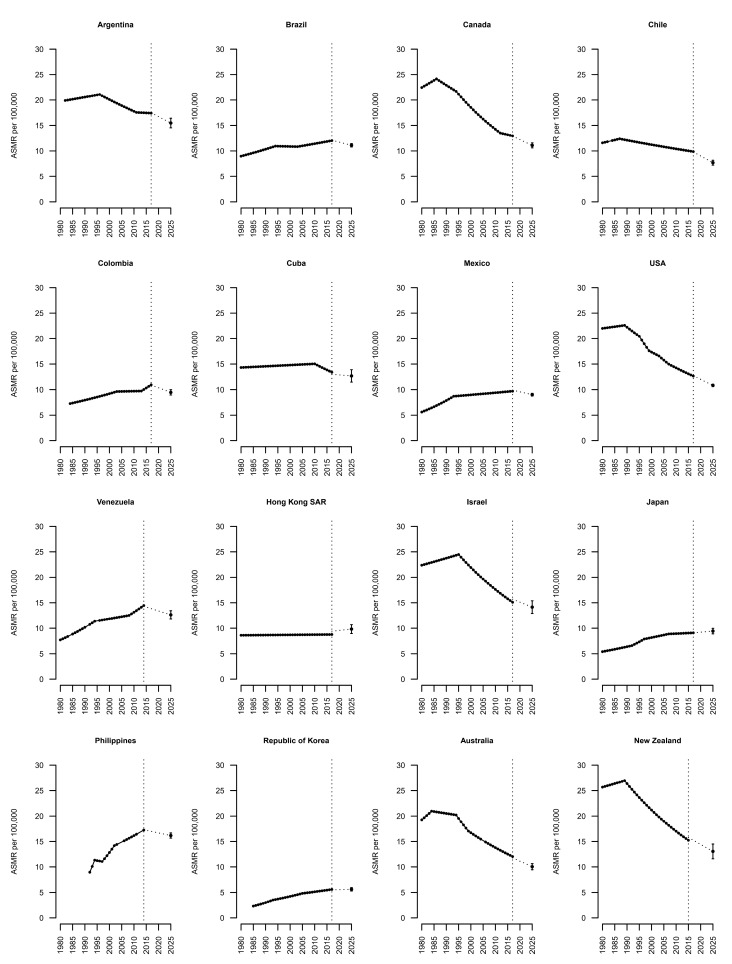
Joinpoint analysis of trends in breast cancer mortality in major countries worldwide from 1980 to 2017 according to data availability.

Long-term joinpoint analysis results, starting from 1980, for the 16 largest American and Australasian countries are presented in Figure 2 and Table 3. Most of the American countries, and particularly in North America, showed stable trends, except for Colombia and Venezuela. In Australia and New Zealand, declining recent trends in breast cancer mortality were observed, which was also true in Israel. The rest of the Asian countries analyzed showed stable trends. In North American countries, we observed a decline in mortality since the 1980s. In Canada, the USA, and Argentina, the mortality rate from breast cancer in the early 1980s was around twice as high as in the other American countries. Most Latin American countries showed unfavorable trends. In Japan and Korea, the trends have been rising, while in Hong Kong the trend has been stable since the 1980s; however, in these countries, the mortality rates for breast cancer remains some of the lowest globally. In Australia, New Zealand, and Israel, the observed trend was firstly upward, although from the 1990s we observed a significant downward trend in mortality.

The number of predicted deaths and the age-standardized mortality rates for the year 2025, as well as comparison figures for 2017, for the selected major countries from North and South America and Australasia are presented in Table 4. We predicted decreased mortality from breast cancer in almost all analyzed countries. Increasing trends were projected in Cuba (4.2%), Hong Kong SAR (4.1%), and Japan (3.1%), while the mortality rates for Asian countries were predicted to remain low on a global scale.

**Figure 3:**
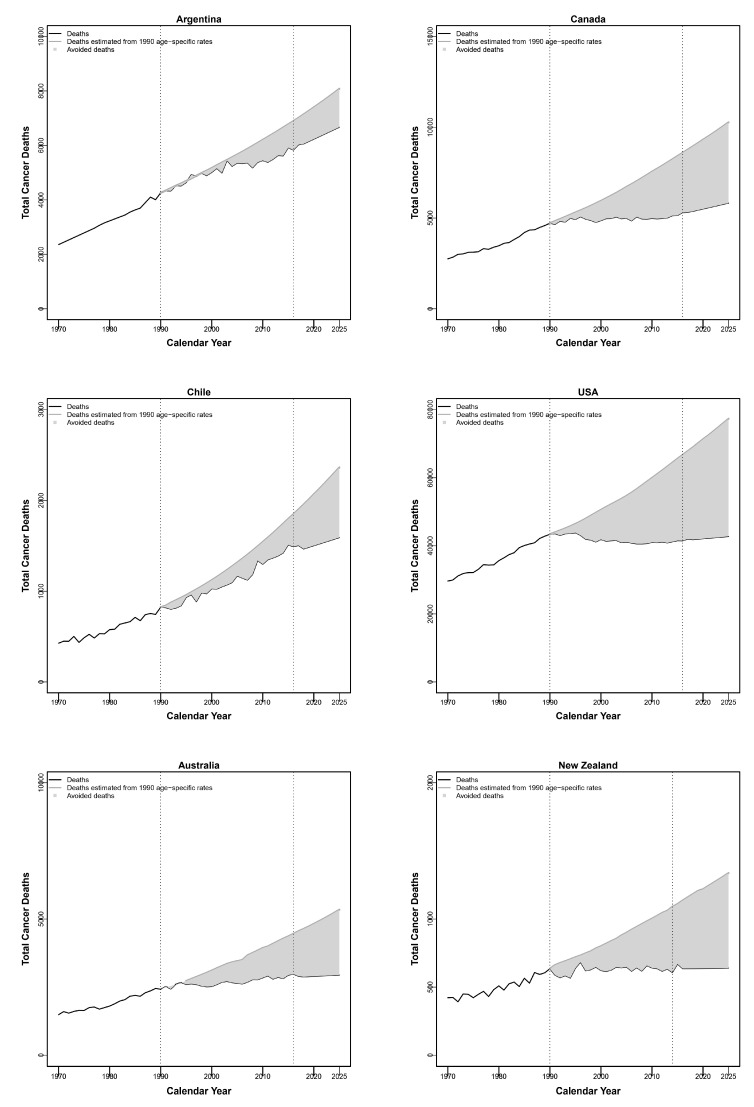
Estimated numbers of avoided breast cancer deaths between 1991 and 2025, assuming constant age-specific rates in 1990 (light grey area).

Figure 3 shows the estimated numbers of avoided breast cancer deaths between 1991 and 2025, assuming constant age-specific rates in 1990 (light gray area). Over the whole period, we estimated a total of about 21,400 avoided breast cancer deaths in Argentina, 77,000 in Canada, 9800 in Chile, 590,000 in the USA, 36,000 in Australia, and 12,000 in New Zealand.

## 4. Discussion

Favorable mortality trends in American and selected Australasian countries are continuing [3]. This decreased mortality is mainly due to the dynamic development of new therapeutic options and progress in the diagnosis of breast cancer. Breast cancer treatments include surgery, radiotherapy, and adjuvant therapy [2]. The first adjuvant therapy consisted of cyclophosphamide, methotrexate, and fluorouracil and was implemented in 1970s [17]. In the following decades, this type of treatment was modified with the use of anthracyclines, taxanes, tamoxifen and subsequently aromatase inhibitors and anti-HER2 agents, which significantly reduce mortality [2,18,19,20,21,22,23].

The subsequent emergence of new therapeutic opportunities has led to differences in the observed trends in breast cancer mortality between races, social classes, and also various areas in the world [24,25]. Advancements have been observed in surgery and radiotherapy over the last few decades [26]. In countries with low and medium Human Development Index (HDI) scores, little improvement in the mortality has been observed. In such countries, breast cancer incidence is lower, although the mortality rate is higher compared to the countries with high HDI scores [1]. The most likely causes include inefficient screening and diagnosis programs and limited access to modern breast cancer therapy. Despite these factors, mortality rates in several Latin American countries remain lower than in North American countries and Oceania. This may be due to higher parity, longer breastfeeding, and a lower prevalence of menopausal hormone therapy [1,27,28,29]. Lifestyle risk factors such as obesity, high alcohol intake, exposure to environmental toxicants and low physical activity are also of considerable importance [1,30]. Difficulties in diagnosis and certification may also partly account for the lower rates.

The breast cancer incidence rates in high-income countries are higher than in most low-income countries. The highest breast cancer incidence rates are observed in Australia, New Zealand, Western and Northern Europe, and North America [1]. Breast cancer incidence rates in these regions tended to increase in the 1980s and 1990s, together with the prevalent use of mammography and consequent overdiagnosis [31,32,33], as well as an increased prevalence of risk factors. Such increases tended to level off in the 2000s, following stable coverage of the population eligible for the screening and reduction in the use of menopausal hormone therapy [34,35,36]. Mammographic screening has also led to decreased incidence of advanced stage breast cancer, contributing to an estimated one-third of the overall decrease in breast cancer mortality [31]. The growing prevalence of obesity influenced the subsequent increase in breast cancer incidence at the beginning of the 21st century in developed countries, as the percentage of ER-positive breast cancer cases increased at that time [1,37,38,39,40,41,42].

Breast cancer incidence is increasing rapidly in low-income countries. This could be related to dynamic lifestyle and sociocultural changes. With the increased participation of women in the labor market in these countries, breast cancer risk factors have increased over recent decades. In these populations, women give birth to their first child later, have fewer children, and breastfeed less often and for shorter periods. Obesity and lower physical activity are of considerable importance as well [1]. An adoption of early diagnosis and screening programs may have a role in incidence trends in low and middle HDI index countries, with consequent effects on mortality, since optimal implementation of early diagnosis and screening may lead to over a 30% reduction in breast cancer mortality [43]. Properly organized screening makes it possible to diagnose cancer at early stages and to initiate appropriate treatment, reducing women’s mortality from breast cancer.

In a previous study, we predicted that the downward mortality trend will continue until 2020 in Canada, the USA, Australia, New Zealand, and Israel [3]. We can now confirm this observation. We also predicted a stable trend for Hong Kong and Japan, which has now been observed. The mortality trend in Korea is still increasing, although at the same time it is still one of the lowest worldwide. In the previous study, we also predicted favorable trends in most Latin American countries. In this paper, we have observed stable recent trends in most Latin American countries.

We predict that in the coming years, in almost all of the analyzed countries there will be changes or continuations of the current trends with decreased breast cancer mortality. Exceptions may be Cuba, Hong Kong, Japan, and Korea, although in these three Asian countries the mortality rates will be still the lowest in the analyzed regions.

We did not take into account the SARS-CoV-2 virus pandemic in our analysis. The first data suggest a negative impact of the pandemic on breast cancer screening and early diagnosis; however, we do not know now what impact this will have on the mortality from this cancer in the coming years [44].

## 5. Conclusions

In most of the analyzed countries in North America, South America, and Asia, we observed stable trends regarding mortality from breast cancer. In the USA, Australia, New Zealand, and Israel, we observed favorable recent trends regarding breast cancer mortality. The epidemiological situation is expected to change in the coming years, which will be reflected in decreased breast cancer mortality in most of the analyzed countries if adequate screening, diagnosis, and treatment are adopted.

## Figures and Tables

**Table 1:** Age-standardized (world population) mortality rates per 100,000 women from breast cancer at all ages in 22 countries ^a^ from the Americas, Asia, and Oceania around 2012 (2010–2014) and in 2017 (or the last available year ^b^), along with deaths from the latest year and the percent changes between the two rates.

All Ages				
Year	Around 2012	2017	Deaths in 2017	% Change
**North America**				
Canada	13.7	12.9	5318	−5.8
USA	13.7	12.7	42,000	−7.3
**Latin America**				
Argentina	17.5	17.5	6049	0.0
Brazil	11.6	12.1	16,724	4.3
Chile	10.2	9.8	1504	−3.9
Colombia	9.8	10.9	3300	11.2
Costa Rica	10.7	10.9	366	1.9
Cuba	14.6	13.0	1519	−11.0
Guatemala	4.8	5.3	353	10.4
Mexico	9.3	9.9	6756	6.5
Panama	10.1	10.2	241	1.0
Puerto Rico	13.0	12.5	445	−3.8
Uruguay	18.4	17.9	611	−2.7
Venezuela	13.8			
**Asia and Oceania**				
Hong Kong SAR	8.5	9.4	721	10.6
Israel	16.9	15.7	1078	−7.1
Japan	9.0	9.1	14,285	1.1
Kuwait	15.2	17.1	102	12.5
Philippines	16.7			
Republic of Korea	5.3	5.5	2497	3.8
Australia	13.3	11.8	2898	−11.3
New Zealand	15.9	15.8	669	−0.6

^a^ Including 14 countries from the Americas and other 8 countries from Asia and Oceania, with over 1 million female inhabitants and mortality data coverage of over 90%, except for Guatemala (88%) and Brazil (87%). ^b^ Last available year for Kuwait is 2015.

**Table 2:** Age-standardized (world population) mortality rates per 100,000 women from breast cancer for 4 age groups in selected countries from the Americas and Australasia around 2012 (2010–2014) and in 2017 (or the last available year ^a^), showing deaths for the latest year and the corresponding percent changes in rates.

Age	Age Group 20–49				Age Group 50–69				Age Group 70–79				Age Group 80+			
Year	2012	2017	Deaths	% Change	2012	2017	Deaths	% Change	2012	2017	Deaths	% Change	2012	2017	Deaths	% Change
**North America**																
Canada	6.1	6.2	488	1.6	42.7	38.5	1944	−9.8	86.9	84.2	1199	−3.1	89.6	88.4	1687	−1.3
USA	6.4	6.1	4139	−4.7	44.4	40.3	17,170	−9.2	84.8	78.7	9293	−7.2	75.8	74.9	11,398	−1.2
**Latin America**																
Argentina	8.3	9.0	838	8.4	58.5	55.4	2298	−5.3	101.1	111.1	1408	9.9	90.1	86.9	1480	−3.6
Brazil	7.1	7.5	3761	5.6	36.9	38.0	7715	3.0	57.5	62.2	2772	8.2	54.7	56.5	2475	3.3
Chile	5.5	4.6	197	−16.4	32.0	31.9	645	−0.3	58.4	60.7	323	3.9	57.7	49.4	339	−14.4
Colombia	5.5	6.0	687	9.1	31.5	35.0	1611	11.1	54.9	59.3	536	8.0	46.7	54.8	464	17.3
Costa Rica	5.2	5.8	63	11.5	34.0	32.7	155	−3.8	62.3	69.9	77	12.2	66.1	62.4	71	−5.6
Cuba	6.5	5.5	163	−15.4	47.8	40.4	595	−15.5	87.3	90.5	355	3.7	84.2	80.4	406	−4.5
Guatemala	3.0	4.2	121	40.0	16.4	15.5	140	−5.5	19.8	29.7	60	50.0	17.9	15.4	31	−14.0
Mexico	5.9	6.2	1795	5.1	31.7	33.8	3280	6.6	42.8	48.9	977	14.3	28.5	29.0	702	1.8
Panama	5.4	6.8	60	25.9	33.5	31.3	102	−6.6	50.9	50.2	39	−1.4	49.3	47.3	40	−4.1
Puerto Rico	6.4	7.0	59	9.4	44.5	37.5	169	−15.7	76.1	88.2	120	15.9	54.4	53.0	97	−2.6
Uruguay	9.0	10.2	75	13.3	59.3	55.7	210	−6.1	113.9	106.1	138	−6.8	96.7	87.8	188	−9.2
Venezuela	7.8				46.9				67.2				57.8			
**Asia and Oceania**																
Hong Kong SAR	4.9	5.6	125	14.3	31.1	35.0	398	12.5	31.4	35.3	83	12.4	62.6	52.3	114	−16.5
Israel	8.1	8.2	142	1.2	52.2	45.4	372	−13.0	102.2	99.8	227	−2.3	227.4	220.3	337	−3.1
Japan	5.3	5.2	1508	−1.9	33.4	33.5	5856	0.3	34.3	38.5	3019	12.2	49.1	55.5	3901	13.0
Kuwait	3.8	2.3	25	−39.5	50.9	33.4	48	−34.4	87.3	129.0	20	47.8	284.3	697.6	7	145.4
Philippines	11.5				56.7				63.9				105.3			
Korea	4.5	4.7	648	4.4	17.2	17.4	1210	1.2	16.1	19.7	367	22.4	20.7	26.3	272	27.1
Australia	6.0	5.1	281	−15.0	42.8	37.1	1074	−13.3	80.3	73.7	635	−8.2	159.4	157.7	908	−1.1
New Zealand	9.2	9.1	95	−1.1	50.6	48.5	272	−4.2	84.0	88.1	134	4.9	160.2	173.8	168	8.5

^a^ Last available year for Kuwait is 2015.

**Table 3:** Joinpoint analysis for breast cancer mortality trends over the 1980–2017 period (according to data availability ^a^).

Country	Trend 1	APC	Trend 2	APC	Trend 3	APC	Trend 4	APC	Trend 5	APC	Trend 6	APC	AAPC
**North America**													
Canada	1980–1986	1.2 ^b^	1986–1994	−1.3 ^b^	1994–2012	−2.6 ^b^	2012–2017	−0.9					−1.5 ^b^
USA	1980–1989	0.3 ^b^	1989–1995	−1.7 ^b^	1995–1999	−3.7 ^b^	1999–2003	−1.5 ^b^	2003–2007	−2.5 ^b^	2007–2017	−1.7 ^b^	−1.5 ^b^
**Latin America**													
Argentina	1982–1996	0.4 ^b^	1996–2011	−1.2 ^b^	2011–2017	−0.1							
Brazil	1980–1994	1.4 ^b^	1994–2003	−0.1	2003–2017	0.7 ^b^							0.8 ^b^
Chile	1980–1987	1	1987–2017	−0.8 ^b^									−0.4 ^b^
Colombia	1984–2003	1.5 ^b^	2003–2013	0.1	2013–2017	3 ^b^							1.2 ^b^
Cuba	1980–2010	0.2 ^b^	2010–2017	−1.6 ^b^									−0.2
Mexico	1980–1993	3.4 ^b^	1993–2017	0.5 ^b^									1.5 ^b^
Venezuela	1980–1994	2.8 ^b^	1994–2008	0.7 ^b^	2008–2014	2.4 ^b^							1.9 ^b^
**Asia and Oceania**													
Hong Kong SAR	1980–2017	0											0
Israel	1980–1995	0.6 ^b^	1995–2017	−2.2 ^b^									−1.1 ^b^
Japan	1980–1992	1.7 ^b^	1992–1997	3.6 ^b^	1997–2007	1.2 ^b^	2007–2017	0.2					1.4 ^b^
Philippines	1992–1994	12.6 ^b^	1994–1997	−0.8	1997–2002	5.1 ^b^	2002–2014	1.6 ^b^					3 ^b^
Korea	1985–1993	5.5 ^b^	1993–2005	2.7 ^b^	2005–2017	1.2 ^b^							2.8 ^b^
Australia	1980–1984	2.1	1984–1994	−0.4	1994–1999	−3.4 ^b^	1999–2017	−1.9 ^b^					−1.3 ^b^
New Zealand	1980–1989	0.5	1989–2015	−2.2 ^b^									−1.5 ^b^

APC: annual percent change; AAPC: average annual percent change. ^a^ Including 16 countries with over 4 million female inhabitants. ^b^ Significantly different from 0 (*p* < 0.05).

**Table 4:** Numbers of predicted deaths and age-standardized mortality rates (ASR) for the year 2025 and comparison figures for 2017 for the selected major countries from the Americas and Australasia, with 95% prediction intervals (PI).

Country	Observed Number of Deaths 2017	Predicted Number of Deaths 2025 (95% PI)	Observed ASR 2017	Predicted ASR 2025 (95% PI)	% Difference 2025 vs. 2017
**North America**					
Canada	5318	5800 (5618–6028)	12.04	11.08 (10.59–11.57)	−8
USA	42,000	42,800 (42,047–43,454)	12	10.82 (10.59–11.06)	−9.8
**Latin America**					
Argentina	6024	6700 (6269–7069)	16.67	15.48 (14.54–16.43)	−7.1
Brazil	16,723	20,100 (19,605–20,605)	11.5	11.1 (10.77–11.43)	−3.4
Chile	1504	1600 (1508–1674)	9.26	7.69 (7.21–8.17)	−16.9
Colombia	3300	3700 (3446–3870)	10.33	9.46 (8.95–9.98)	−8.4
Cuba	1519	1700 (1621–1870)	12.17	12.68 (11.47–13.89)	4.2
Mexico	6755	8100 (7845–8308)	9.64	9.02 (8.76–9.29)	−6.4
Venezuela	2204	2700 (2579–2917)	13.69	12.64 (11.84–13.43)	−7.7
**Asia and Oceania**					
Hong Kong SAR	720	800 (771–919)	9.44	9.83 (8.96–10.69)	4.1
Israel	1078	1100 (1011–1191)	15.75	14.15 (12.9–15.39)	−10.2
Japan	14,284	16,200 (15,697–16,752)	9.15	9.43 (8.9–9.97)	3.1
Philippines	7145	9600 (9270–9848)	17.25	16.17 (15.67–16.67)	−6.3
Korea	2497	2800 (2635–2913)	5.53	5.62 (5.28–5.95)	1.6
Australia	2898	2900 (2797–3092)	11.76	10.04 (9.44–10.64)	−14.7
New Zealand	669	600 (578–703)	15.78	13.07 (11.62–14.51)	−17.2

## Data Availability

The data presented in this study are available in this article.
